# A basic helix-loop-helix transcription factor, *PhFBH4*, regulates flower senescence by modulating ethylene biosynthesis pathway in petunia

**DOI:** 10.1038/hortres.2015.59

**Published:** 2015-12-16

**Authors:** Jing Yin, Xiaoxiao Chang, Takao Kasuga, Mai Bui, Michael S Reid, Cai-Zhong Jiang

**Affiliations:** 1 Department of Ornamental Horticulture, China Agricultural University, Beijing 100193, China; 2 Department of Plant Sciences, University of California, Davis, One Shields Avenue, Davis, CA 95616, USA; 3 Department of Horticulture, Northwest A&F University, Yangling, Shanxi, China; 4 Crops Pathology and Genetic Research Unit, United States Department of Agriculture, Agricultural Research Service, One Shields Avenue, Davis, CA 95616, USA

## Abstract

The basic helix-loop-helix (bHLH) transcription factors (TFs) play important roles in regulating multiple biological processes in plants. However, there are few reports about the function of bHLHs in flower senescence. In this study, a bHLH TF, *PhFBH4*, was found to be dramatically upregulated during flower senescence. Transcription of *PhFBH4* is induced by plant hormones and abiotic stress treatments. Silencing of *PhFBH4* using virus-induced gene silencing or an antisense approach extended flower longevity, while transgenic petunia flowers with an overexpression construct showed a reduction in flower lifespan. Abundance of transcripts of senescence-related genes (*SAG12*, *SAG29*) was significantly changed in petunia *PhFBH4* transgenic flowers. Furthermore, silencing or overexpression of *PhFBH4* reduced or increased, respectively, transcript abundances of important ethylene biosynthesis-related genes, *ACS1* and *ACO1*, thereby influencing ethylene production. An electrophoretic mobility shift assay showed that the PhFBH4 protein physically interacted with the G-box *cis*-element in the promoter of *ACS1,* suggesting that *ACS1* was a direct target of the PhFBH4 protein. In addition, ectopic expression of this gene altered plant development including plant height, internode length, and size of leaves and flowers, accompanied by alteration of transcript abundance of the gibberellin biosynthesis-related gene *GA2OX3*. Our results indicate that *PhFBH4* plays an important role in regulating plant growth and development through modulating the ethylene biosynthesis pathway.

## Introduction

Flower senescence is an important coordinated process regulated by internal and environmental changes.^1^ Microarray studies of gene expression have been used to generate genome-wide transcriptome profiles of senescing petals in *Arabidopsis*.^2^ The data revealed that hundreds of upregulated and downregulated genes, including various transcription factors (TFs), are involved in flower senescence progress. TFs play critical roles in plant growth and development. The most represented families amongst TFs specifically upregulated during petal senescence in *Arabidopsis* were AP2-EREBP, homeobox (HB), and AUX-IAA.^2^ The upregulation of the AP2-EREB TFs establishes the role of ethylene in *Arabidopsis*. In petunia flowers, expression profiles of the ethylene-responsive element-binding factor (ERF) family genes were studied in detail.^3^ Some of ERFs appear to be associated with fruit ripening and with corolla senescence.^4–7^ Among the HB TFs upregulated in *Arabidopsis* petals was KNAT1, a member of the Class I KNOX family known to modulate cytokinin levels.^8^ The expression of genes encoding AUX-IAA proteins in *Arabidopsis* suggests the role of auxin in petal senescence. Nevertheless, exact roles of these regulatory elements in the process of flower senescence are still largely unknown.

Petunia is an ideal model system for studies of flower senescence due to its short life cycle, vast genetic resources, and large number of amenities for biochemical and molecular analysis.^9^ We identified a cluster of genes upregulated during development and senescence of petunia flowers, including several transcription factors.^10,11^ In addition, we have successfully employed tobacco rattle virus (TRV)-based virus-induced gene silencing (VIGS) to study the function of senescence-related genes in petunia corollas.^12,13^ By microarray analysis, we previously determined that a homeodomain-leucine-zipper TF, PhHD-ZIP was upregulated during flower senescence.^10^ Silencing *PhHD-Zip* using VIGS resulted in extended flower longevity. Transcript abundances of ethylene biosynthesis-related genes and ethylene production were dramatically reduced in the *PhHD-Zip*-silenced flowers. On the other hand, overexpression of *PhHD-Zip* in petunia caused early flower senescence. Furthermore, *PhHD-Zip* transcript levels in petunia flower were increased by hormones (ethylene, ABA) and abiotic stresses (dehydration, NaCl, and cold). The results suggest that PhHD-Zip plays an important role in regulating petunia flower senescence.^11^ From the same microarray study,^10^ a basic helix-loop-helix (bHLH) TF was also found to be highly expressed in flower petals and upregulated during the flower senescence process, suggesting that it may also play a role in the regulation of flower senescence.

The bHLH TFs constitute a large family of regulatory proteins found in plants and animals. One hundred and sixty two genes in *Arabidopsis* and 167 genes in rice have been predicted to encode bHLHs.^14–16^ These proteins have a characteristic, highly conserved bHLH domain and comprise two functionally distinct regions comprising approximately 60 amino acids.^17^ The basic region, located at the N-terminal end of the domain, consists of 13–17 primarily basic amino acids and is involved in DNA binding. The HLH region, at the C-terminal end, is rich in hydrophobic residues and contributes to the formation of homodimers or heterodimers.^18,19^ Outside the conserved bHLH domain, these proteins exhibit considerable sequence divergence.^20^ So far, functional analysis shows that bHLH proteins play important roles in fruit dehiscence, epidermal cell development, flavonoid biosynthesis, phytochrome signaling, plant hormone signaling, and biotic/abiotic stress responses.^18,21–26^ For example, FLOWERING bHLH 1 (FBH1), FBH2, FBH3, and FBH4 bind to the E-box *cis*-elements in the *CONSTANS* (*CO*) promoter. Overexpression of all FBH genes increased *CO* expression levels and resulted in early flowering.^27^ However, there has previously been no report of a function for bHLH TFs in flower senescence.

Here, we report the functional characterization of a bHLH TF, *PhFBH4*. Petunia plants in which bHLH expression was downregulated by VIGS or antisense silencing showed extended flower longevity while overexpression of the *PhFBH4* in petunia resulted in earlier flower senescence. These data suggest an important role for PhFBH4 in the control of flower senescence.

## Materials and methods

### Plant material

*Petunia hybrida* cv. ‘Primetime Blue’ was used for VIGS experiments. Four-week-old seedlings were transferred to pots and used for VIGS inoculation. After inoculation, the seedlings were placed in a growth chamber with a day (16 h)/night (8 h) cycle and temperature regime of 25/20 °C. *Petunia hybrida* cv. ‘Mitchell diploid’ was used for stable transformation. Wild-type (WT) and transgenic plants were grown under standard greenhouse conditions.

### Hormone and abiotic stress treatments

Hormone and abiotic stress treatments were carried out as described in detail by Chang *et al*.^11^ Briefly, the whole flowers were harvested from the plant prior to anther dehiscence. For each treatment, at least three individual flowers were used. The harvested flowers were immediately placed in 2-ml microtubes with or without water, 0.1 mM ABA, 50 µM GA_3_, or 100 mM NaCl at room temperature. For low and high temperature treatments, harvested flowers were placed in the tubes with water and treated in a 4 °C cold room or in a 29 °C room. For ethylene treatment, flowers were placed in tubes with water, then sealed in a large glass container and treated with 3 µl/l ethylene. Corollas were collected at 0, 3, 6, and 12 h after treatment. For 1-methylcyclopropene (1-MCP) treatment, flowers were first treated with 50 nl/l 1-MCP for 4 h, and then exposed to 3 µl/l ethylene. Corollas were collected at 0, 3, 6, and 12 h after the ethylene treatment. Harvested tissues were immediately frozen in liquid nitrogen, and then kept at –80 °C until RNA extraction.

### VIGS vector construction and petunia inoculation

The chalcone synthase (CHS)/TRV2 construct was generated previously.^28^ To generate the PhFBH4/CHS/TRV2 construct, a 326 bp DNA fragment was amplified from petunia cDNA with gene-specific primers (Supplementary Table S1) and then cloned into the CHS/TRV2 vector as described previously by Chen *et al*.^12^ and Chang *et al*.^11^ *Agrobacterium tumefaciens* strain GV3101 was transformed with the VIGS constructs. The presence of the gene fragments in the bacteria was confirmed by polymerase chain reaction (PCR) with gene-specific primers.

Four-week-old plants (when the first 4 true leaves had emerged) were used for VIGS inoculation following the protocol that has been previously described by Jiang *et al*.^13^

### Plasmid construction and stable transformation of petunias

To generate *PhFBH4* silencing transgenic plants, a 281 bp fragment was cloned into the BamHΙ and SpeΙ site of the pGSA1403 vector in the antisense orientation. To generate *PhFBH4* overexpression transgenic plants, a 1056 bp DNA sequence containing the ORF region of *PhFBH4* gene was cloned into the BamHΙ and SpeΙ site of the pGSA1403 vector in the sense orientation. The constructs were transformed into *A. tumefaciens* strain LBA4404 using electroporation. PCR amplification was performed to confirm the destination vectors had integrated with the *PhFBH4* gene.

Transformation and regeneration of petunia ‘Mitchell Diploid’ was performed using the leaf disk method and cultivation process described previously by Wang *et al.*^10^ At least 12 independent transgenic lines were generated. Homozygous lines from T2 or later generations were used for later experiments.

### Flower longevity

Flower longevity was recorded as the time from anthesis until the corolla was completely wilted.^11^ At least 10 flowers from different plants were used for longevity studies. Data were statistically analyzed using JMP10.0 software package (SAS Institute, Cary, NC, USA).

### RNA extraction, semi-quantitative, and quantitative RT-PCR

Petals of wild–type (WT) and transgenic flowers under the different treatments were collected. Total RNA was extracted from petunia corollas using Trizol Reagent according to the manufacturer’s instructions (Invitrogen, Carlsbad, CA, USA). RNA was treated with RNase-free DNase (Promega, Madison, WI, USA) to remove DNA contamination according to the manufacturer’s instructions. Two micrograms of total RNA was used to synthesize first-strand cDNA using the SuperScript III kit (Invitrogen). Semi-quantitative RT-PCR was performed in 25 μl reactions containing 0.3 μM of each primer and 1 μl of template cDNA. Amplification by qPCR was performed in 25 μl reactions containing 1 μl cDNA, 0.3 μM of each primer, and 12.5 μl of SYBR Green PCR Master Mix (Toyobo, New York, NY, USA). 26S ribosomal RNA served as an internal control.^10,12^

### Measurement of ethylene production

Ethylene production was measured as described previously by Chang *et al*.^11^ Briefly, at 5 days after anthesis (D5), each individual flower was placed in a tube with water and incubated for 3 h at 25 °C. A 1 ml sample of gas was collected using a gas-tight hypodermic syringe and injected into a gas chromatographer (GC-8A; Shimadzu, Kyoto, Japan) for ethylene detection and measurement. Five biological replicates were used for each measurement.

### Electrophoretic mobility shift assay

The electrophoretic mobility shift assay (EMSA) was performed according to Luo *et al*.^29^ Recombinant pGEX-PhFBH4 proteins were produced in *Escherichia coli* strain BL21. The *E. coli* cells were lysed by sonication and purified with glutathione–Sepharose 4B beads (GE Healthcare, Sunnyvale, CA, USA). The proACO1 probe 5′-TTAAATCACACATGAATAATATCAAATGTTTTGGTC-3′, the proACS1 probe 5′-TTTCTTTCTCACGTGTAGCTTCTA-3′, the mutated proACS1 probe 5‘-TTTCTTTCTGCTACTTAGCTTCTA-3’ and their complementary probes were labeled with biotin. For each binding reaction, 1 μg aliquot recombinant protein and 2 nM biotin-labeled probe were used. The LightShift chemiluminescent EMSA kit (Pierce, Rockford, IL, USA) was used for detection.^29^

## Results

### Isolation and expression pattern of PhFBH4

One transcript, annotated as bHLH TF, was identified among genes upregulated during flower senescence using a custom Nimblegen microarray (Supplementary Figure S1).^10^ To analyze the function of this putative transcription factor, full-length cDNA was cloned by rapid amplification of cDNA ends. Sequence analysis showed that this TF encodes 345 amino acids with a bHLH domain. Phylogenetic analysis suggested that this bHLH protein belongs to the bHLH IX family^17^ and has a high homology with AtFBH4, SlFBH4, and StFBH4 (Supplementary Figure S2). Therefore, the gene was named *PhFBH4*.

**Figure 1:**
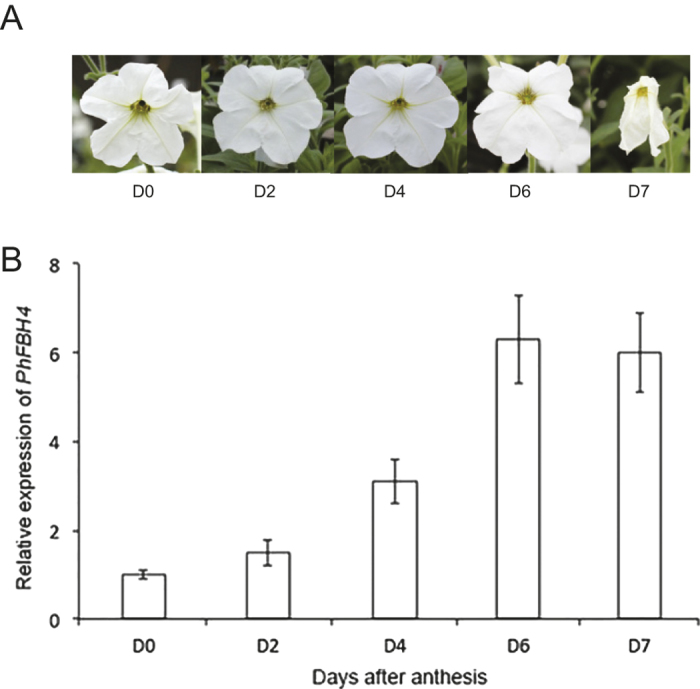
*PhFBH4* transcripts in different flower stages. (**A**) Different stages of petunia flower after anthesis. D0: the day of anthesis, D2, D4, D6, D7: 2, 4, 6, and 7 days after anthesis, respectively. (**B**) Expression level of *PhFBH4* in different flower stages by qRT-PCR. Error bars show SD of the means of three biological replicates.

To confirm the microarray data, flowers at different stages were collected (Figure 1A) and qRT-PCR was used to detect the expression level of *PhFBH4*. The *PhFBH4* transcript level constantly increased in petals from the just fully opened flower (D0) to early wilted flower (D6) and then remained high until wilting completed (D7; Figure 1B). Furthermore, *PhFBH4* transcripts were detected in the leaf, stem, root, and all flower organs (Supplementary Figure S3). One interesting feature of *PhFBH4* is that expression level in old leaves is much higher than that in young leaves.

### PhFBH4 expression is regulated by ethylene, ABA and abiotic stresses

**Figure 2:**
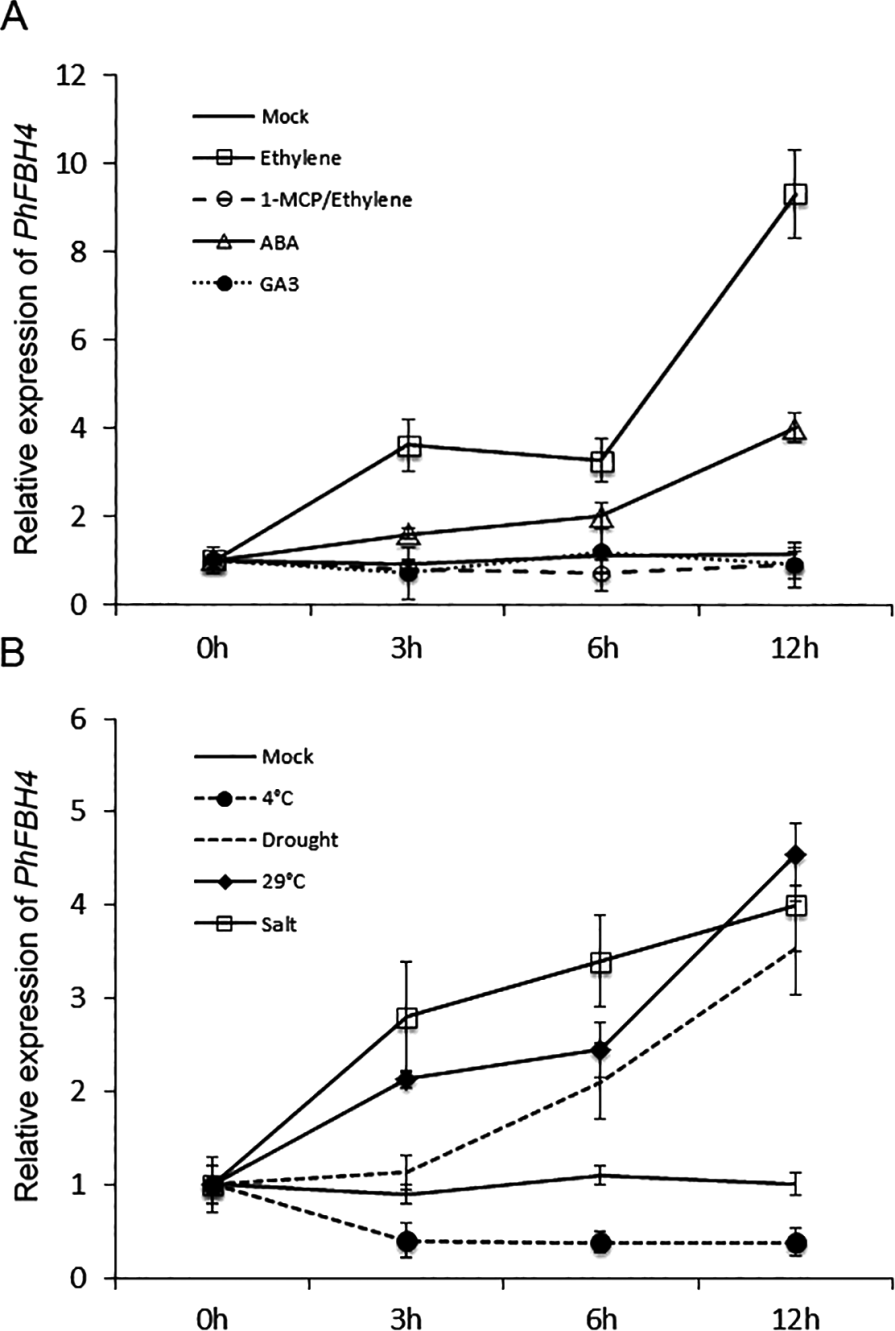
Expression pattern of *PhFBH4* under hormone and abiotic treatment by qRT-PCR. (**A**) Expression of *PhFBH4* under hormone treatment. Petunia flowers harvested at anthesis were placed in tubes with water (mock), 0.1 mM ABA, 50 μM GA3, treated with ethylene (3 µl/l), or with 1-MCP for 4 h before ethylene treatment. (**B**) Expression of *PhFBH4* under abiotic stress treatment. Petunia flowers were placed in tubes with water at 29 °C and 4 °C, without water (drought), or with 100 mM NaCl at room temperature (salt). Error bars show SD of the means of three biological replicates.

As hormones and stresses play important roles in flower senescence, we determined the expression pattern of *PhFBH4* in petals after different hormone and abiotic stress treatments. Flowers treated with ethylene, ABA, drought, high temperature, and salt displayed higher expression of *PhFBH4* than untreated controls (Figure 2). Cold treatment inhibited *PhFBH4* expression by 50% after 3 h. Application of GA_3_ did not affect *PhFBH4* expression (Figure 2).

### Virus-induced PhFBH4 silencing extended flower longevity

**Figure 3:**
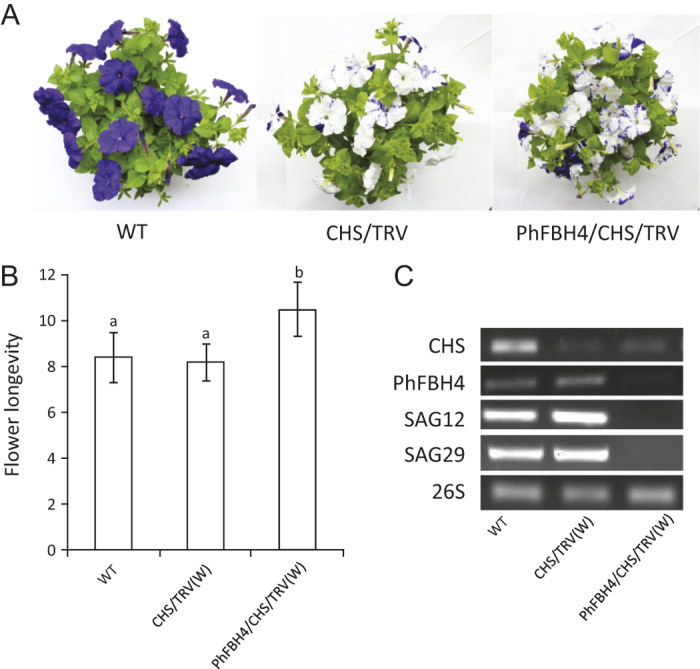
Silencing *PhFBH4* using the VIGS system extended flower longevity. (**A**) Phenotype of plants using different constructs at 5 weeks after inoculation. (**B**) Flower longevity of attached flowers in different plants. CHS/TRV (W), white flowers in CHS/TRV plants; PhFBH4/CHS/TRV (W), white flowers in PhFBH4/CHS/TRV plants. Error bars indicate SD (*n* ≥ 10). Different letters denote significant differences at *p* > 0.05 analyzed by Tukey's test (**C**) Abundance of related gene expression in flowers on D7 in different plants.

The TRV-based VIGS system has proven to be an efficient and fast method to silence target genes in petunia.^12^ Therefore, to quickly study the function of *PhFBH4*, a 308 bp fragment of *PhFBH4* was cloned into a silencing construct bearing a fragment of the petunia *CHS* gene as a visual reporter. Five weeks after inoculation, white sectors were observed on the normally purple corollas indicating silencing of *CHS* (Figure 3A). Semi-quantitative RT-PCR was used to detect the gene expression in different plants. Compared with WT and *CHS/TRV* controls, the *PhFBH4* gene transcript was clearly downregulated in the white flowers of the *PhFBH4/CHS/TRV* plants (Figure 3C). Silencing *PhFBH4* extended flower longevity by two more days in comparison to the controls (WT flowers and white flowers from CHS/TRV) (Figure 3B). Transcripts of the senescence marker genes, *SAG12* and *SAG29*, were barely detected in white flowers of *PhFBH4/CHS/TRV* plants 7 days after anthesis, indicating senescence progress was delayed by silencing *PhFBH4* (Figure 3C).

### PhFBH4 regulates flower senescence by modulating ethylene biosynthesis

**Figure 4:**
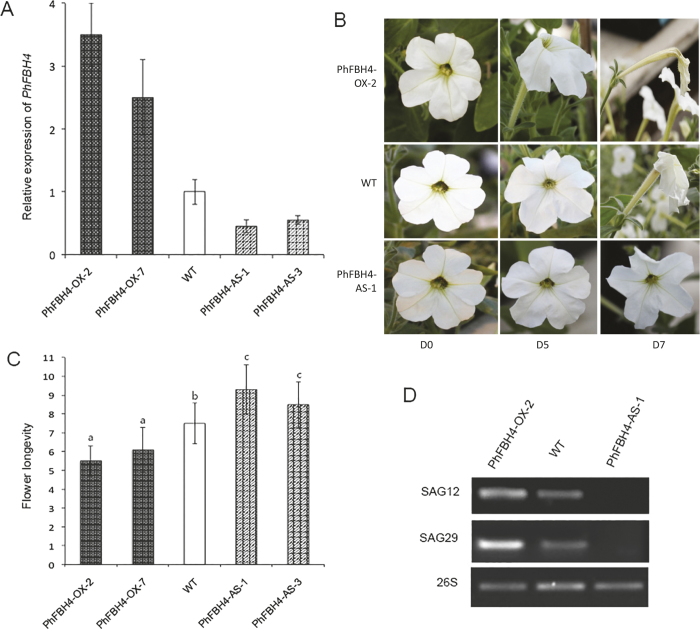
Ectopic expression of *PhFBH4* affected flower longevity. (**A**) Expression of *PhFBH4* in WT, *PhFBH4* overexpression and antisense silencing transgenic plants by qRT-PCR. PhFBH4-OX-2, PhFBH4-OX-7, different lines of *PhFBH4* overexpression. PhFBH4-AS-1, PhFBH4-AS-3, different lines of *PhFBH4* antisense silencing. Error bars show SE of the means of three biological replicates. (**B**) Different flower stages in WT petunia and *PhFBH4* transgenic petunia. D0: the day of anthesis; D5, D7: 5 and 7 days after anthesis, respectively. (**C**) Flower longevity in WT and *PhFBH4*-OX and AS transgenic plants. Error bars indicate SD (*n* ≥ 10). Different letters denote significant differences at *p* ≤ 0.05 analyzed by Tukey's test. (**D**) Senescence marker genes, *SAG12* and *SAG29*, expression in the flower of wide type petunia and transgenic petunia on D5.

To further investigate the function of *PhFBH4*, we generated *PhFBH4* overexpression and antisense silencing transgenic plants in petunia. Expression level of *PhFBH4* was determined by qRT-PCR (Figure 4A). To confirm whether *PhFBH4* is involved in flower senescence, the flower longevity of WT and transgenic plants was recorded (Figure 4B). The longevity of intact WT flowers was approximately 7 days. Overexpression of *PhFBH4* accelerated flower senescence and shortened flower longevity to 5.5 days, while silencing of *PhFBH4* extended flower longevity to about 9 days (Figure 4C). Since petunia plants of *PhFBH4-OX-2* and *PhFBH4-AS-1* exhibited the strongest expression and flower longevity difference, they were chosen for further analysis. Transcript abundance of the senescence marker genes, *SAG12* and *SAG29*, were correlated with the flower longevity of WT and transgenic plants (Figure 4D).

**Figure 5:**
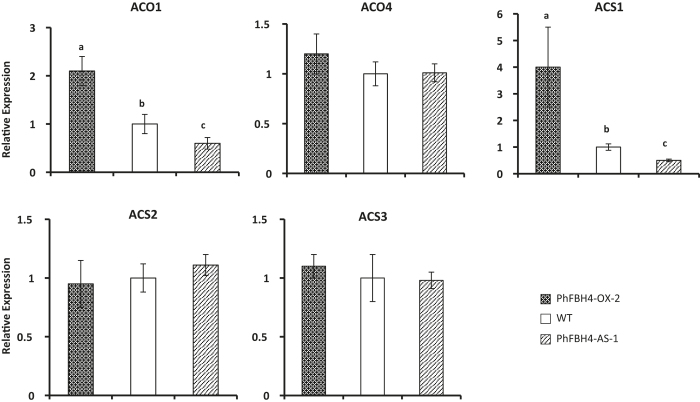
Expression of ethylene biosynthesis-related genes in D5 petunia flowers. Abundance of transcripts of genes associated with ethylene biosynthesis was determined at D5 in WT and *PhFBH4* transgenic plants. Error bars show SD of the means of three biological replicates. Different letters denote significant differences at *p* ≤ 0.05 analyzed by Tukey's test.

**Figure 6:**
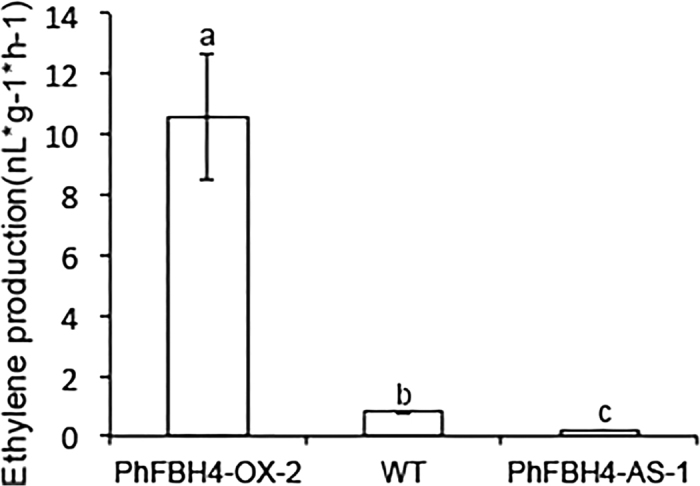
Effect of ectopic expression of *PhFBH4* on ethylene production of petunia flowers in WT and transgenic plants. Ethylene production was measured at D5. Error bars show SD of the means of five biological replicates. Different letters denote significant differences at *p* ≤ 0.05 analyzed by Tukey's test.

As ethylene is important in flower senescence, we then investigated the abundance of *ACO* and *ACS* gene transcripts and ethylene production in WT and transgenic plants. *ACO1* and *ACS1* transcript levels were significantly upregulated in *PhFBH4-OX* plants compared with those of WT. On the other hand, *ACO1* and *ACS1* expression levels were decreased in *PhFBH4-AS* plants (Figure 5). Ethylene production further confirmed the transcriptional differences. Ethylene production on day 5 was significantly reduced in the *PhFBH4* silenced flowers and was much higher in *PhFBH4* overexpression flowers than that in WT flowers (Figure 6). These results suggested that *PhFBH4* regulates flower senescence by mediating the ethylene biosynthetic genes *ACO1* and *ACS1.*

**Figure 7:**
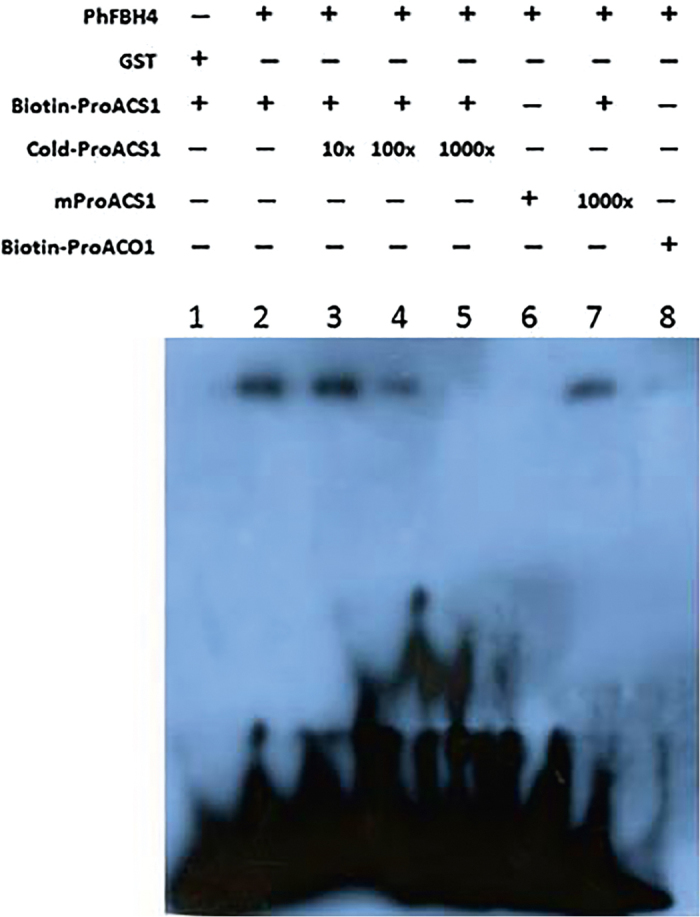
Electrophoretic mobility shift assay of the PhFBH4 protein. The biotin-labeled oligonucleotide of *proACS1* was mixed with GST-tagged protein control (lane 1) or PhFBH4 proteins prepared from cells transfected with *pGST::PhFBH4* plasmid (lanes 2–7). PhFBH4 protein physically binds a *cis*-element (G-box) in the *PhACS1* promoter (lane 2). Binding was gradually abolished by the addition of an unlabeled oligonucleotide competitor in 10-fold (lane 3), 100-fold (lane 4), and 1000-fold (lane 5) molar excess, whereas specific binding was maintained when the same amount of the excess mutant oligonucleotide competitor was added (lanes 6 and 7). PhFBH4 protein failed to bind the E-boxes in the promoter of *ACO1* (lane 8).

To test the direct binding between PhFBH4 protein and promoters of *ACO1/ACS1*, an EMSA was carried out. A search for potential PhFBH4-binding motifs revealed the presence of the E-box motif CANNTG and the G-box motif CACGTG in the 1.5 kb-upstream promoter regions of the petunia *ACS1* and *ACO1* genes. The biotin-labeled probes were designed to bind to the E/G-box element in the promoter of *ACO1* and *ACS1* (Figure 7). The EMSA result showed that the PhFBH4 protein was capable of binding to the biotin-labeled probe of the G-box in the promoter of *ACS1* (Figure 7, lane 2). Binding was gradually abolished by the addition of an unlabeled oligonucleotide competitor in 10-fold (lane 3), 100-fold (lane 4), and 1000-fold (lane 5) molar excess. No binding was observed when mutant oligonucleotide probes were used (lane 6), whereas specific binding was maintained when the same amount of the excess mutant competitor was added (lane 7). However, there was no binding between the PhFBH4 protein and the E-boxes in the promoter of *ACO1* (Figure 7, lane 8). These results suggest that *ACS1* is a direct target of PhFBH4.

### Ectopic expression of PhFBH4 affects plant growth

**Figure 8:**
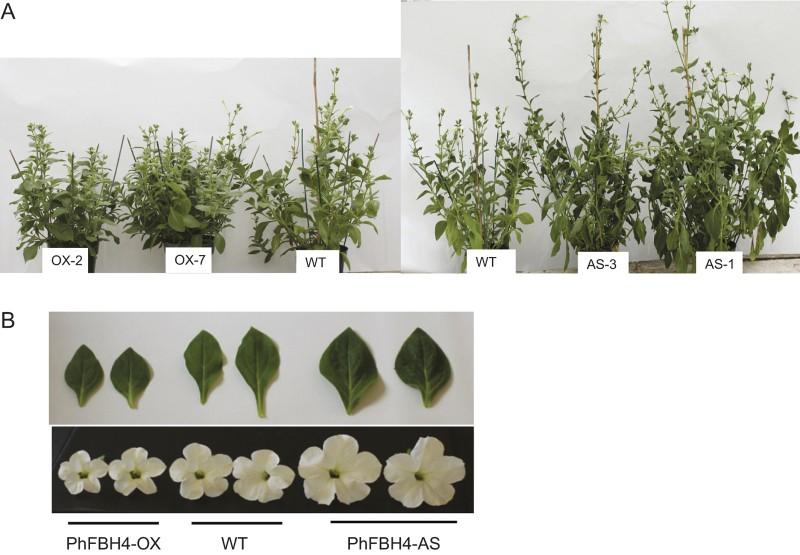
Phenotypic traits of the *PhFBH4* overexpression (OX) and antisense silencing (AS) transgenic petunia plants. (**A**) Whole plants of different lines. (**B**) Leaf size and flower size of different plants. Flowers were harvested on D4. Leaves were collected at the sixth from the top.

At 50 days after transplanting, phenotypical differences were clearly observed between WT and transgenic plants (Figure 8). Leaves and flowers of *PhFBH4-AS* plants were significantly larger than WT control while leaves and flowers of *PhFBH4-OX* plants were smaller (Figure 8). Changes in PhFBH4 expression also affected plant height. Stem and internode lengths of the antisense transgenic lines were significantly greater than those of the controls, and were significantly smaller in the over-expressing transgenic lines (Table 1). There were no differences in the number of internodes or flowering time between WT and transgenic plants (Table 1).

**Figure 9:**
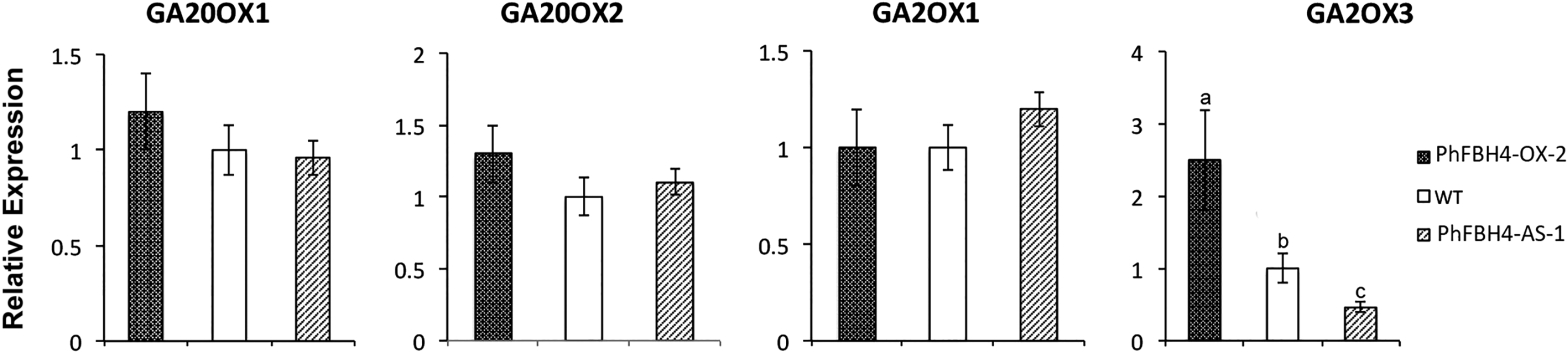
Expression analysis of GA-related genes in the flower of WT and transgenic petunia on D4 by qRT-PCR. Abundance of transcripts of genes associated with GA was determined at D4 in WT and *PhFBH4* transgenic plants. Error bars show SD of the means of three biological replicates. Different letters denote significant differences at *p* ≤ 0.05 analyzed by Tukey's test.

The plant hormone gibberellin (GA) plays an important role in many aspects of plant development, particularly in plant height and stem elongation.^30^ To test whether the phenotypic differences between WT and *PhFBH4* transgenic plants were associated with the GA pathway, transcript abundances of GA biosynthesis-related genes were determined. qRT-PCR results showed that the transcript abundances of the GA biosynthetic genes, *GA20ox1 and GA20ox2*, were not changed in WT and *PhFBH4* transgenic plants. However, one of the GA metabolic genes *GA2ox3* displayed a significant difference in abundance. The transcriptional level of *GA2ox3* in *PhFBH4-OX* plants was increased 2.74-fold and was reduced 48% in *PhFBH4-AS* plants, compared to that of WT controls (Figure 9). The significant difference in *GA2ox3* transcript levels could result in a change of bioactive GA, thereby influencing the growth in *PhFBH4* transgenic plants.

## Discussion

### PhFBH4 is involved in flower senescence by modulating ethylene biosynthesis

In this study, we identified a bHLH TF, named *PhFBH4* because of its sequence similarity to *Arabidopsis FBH4*. We found that its transcript abundance increased dramatically during flower senescence, and increased in response to a range of abiotic stressors and to treatments with plant hormones, particularly ethylene. The gaseous phytohormone ethylene is known to play a critical role in flower senescence.^31^ In many flowers, the onset of floral senescence is initiated by a climacteric rise in ethylene production.^32^ Application of ethylene accelerates flower senescence, while ethylene inhibitors such as 1-MCP can significantly delay the senescence process.^33–35^ The biosynthetic pathway of ethylene has been well studied.^36^ Two important enzymes, 1-aminocyclopropane-1-carboxylate synthase (ACS), which catalyzes the conversion of *S*-adenosylmethionine (AdoMet) to ACC, and ACC oxidase (ACO), which converts ACC into ethylene, are encoded by multiple gene families.^37^ In carnation, petunia, and tomato, the increase in the ethylene synthesis is accompanied by increased *ACS* and *ACO* gene expression and elevated enzyme activities.^38–41^ Downregulation of the *ACO* gene in carnation and petunia causes low ethylene production and markedly delayed petal senescence.^12,42^ However, the transcriptional regulation of the ethylene biosynthesis pathway during the flower senescence has been little studied. In this study, manipulation of *PhFBH4* expression using overexpression and silencing approaches altered flower longevity, accompanied by alterations in the transcript abundances of ethylene biosynthesis genes *ACO1* and *ACS1* and ethylene production. Furthermore, our results in the EMSA suggest that PhFBH4 directly binds to the promoter of *ACS1* (Figure 7). Our data demonstrates that PhFBH4 is involved in the regulation of flower senescence progress through its interaction with the ethylene biosynthesis pathway.

In *Arabidopsis thaliana*, FLOWERING BHLH 1 (FBH1), FBH2, FBH3, and FBH4 were identified as four *CO* transcriptional activators. All FBH proteins are related to the bHLH-type TFs that preferentially bind to the E-box *cis*-elements in the *CO* promoter. Overexpression of all *FBH* genes caused early flowering regardless of photoperiod. Furthermore, *FBH* homologs in poplar and rice induced *CO* expression in *Arabidopsis*.^27^ However, the early flowering phenotype seen in *Arabidopsis FBH-OX* was not observed in *PhFBH4-OX* transgenic petunia plants. Interestingly, PhFBH4 only binds the G-box *cis*-elements in the *ACS1* promoter. This may explain the difference in flowering timing between *Arabidopsis* and petunia.

### PhFBH4 may be involved in crosstalk between ethylene and GA during plant development

In addition to its positive role in flower senescence, ethylene is generally considered a growth inhibitor.^43^ After ethylene treatment, rapid inhibition of elongation was reported in stems of *Pisum sativum*,^44^ leaves of *Poa* species^45^ and roots of *Cucumis sativus*.^46^ Ethylene overproduction reduces internode length through modification of ACS and ACO gene expression levels in *Nicotiana tabacum*.^47^

Gibberellins and ethylene are both involved in the control of plant developmental processes from seed germination and cell elongation in hypocotyls to formation of stomata and flower senescence.^48,49^ Crosstalk between GAs and ethylene, as well as with other hormones, has been demonstrated in *Arabidopsis*. Bioactive GA levels are low in the *ctr1* mutant and after ACC treatment but increase in the ethylene-insensitive *etr1-2* mutant.^50–52^ Furthermore, ethylene regulates the maintenance and exaggeration of the apical hook by modifying DELLA degradation.^53^ Active ethylene signaling results in decreased GA content, thus stabilizing DELLA proteins.^51^ Recent evidence suggests that reduction in the bioactive GA content enhances ethylene-mediated flower senescence in rose.^54^ These results suggest that the antagonism effect between ethylene and GA is mediated by regulating bioactive GA levels and the stability of DELLA proteins.^55^ In carnation, exogenous application of GA can delay the senescence of cut flowers by reducing ethylene production.^56^ In this study, overexpression of *PhFBH4* increased the abundance of transcripts of ethylene biosynthesis genes (*ACO1/ACS1*; Figure 5) and also increased ethylene production (Figure 6). Moreover, the increased expression of the GA metabolic gene *GA2ox3* in *PhFBH4-OX* transgenic plants would raise bioactive GAs content, while silencing *PhFBH4* would reduce their levels (Figure 9). Our data support this hypothesis. In *PhFBH4-OX* transgenic plants, which produced more ethylene and would hypothetically have less bioactive GA than WT petunia, we observed reduced stem, leaf and flower size. In contrast, silencing of *PhFBH4* resulted in longer internode length and larger leaves and flowers compared with WT. Furthermore, overexpression of *PhFBH4* accelerated flower senescence and shortened flower longevity, while silencing of *PhFBH4* extended flower longevity (Figure 4C). These results suggest that PhFBH4 mediates an antagonistic relationship of ethylene and GA in plant growth and flower senescence. It would be interesting to comprehensively analyze the relationships between plant growth and concentrations of some of bioactive GAs or expression levels of expanded GA-related genes such as *GA3ox* genes in the future.

## Figures and Tables

**Table 1:** Phenotypic characteristics of WT and *PhFBH4* transgenic plants.

	PhFBH4-OX-2	PhFBH4-OX-7	WT	PhFBH4-AS-1	PhFBH4-AS-3
Height	45.3^a^ ± 3.7	51.2^ab^ ± 3.2	54.7^b^ ± 3.5	67.8^c^ ± 4.1	60.2^cd^ ± 3.9
Number of internode	17.2 ± 2.1	17.4 ± 2.8	16.5 ± 2.3	17.5 ± 2.7	17.1 ± 2.4
Mean internode length	2.63^a^ ± 0.38	2.93^ab^ ± 0.32	3.31^b^ ± 0.47	3.87^c^ ± 0.24	3.52^b^ ± 0.26
Days until first flower	77.2 ± 1.4	78.4 ± 2.1	78.1 ± 1.4	77.6 ± 1.8	77.6 ± 1.2
Flower diameter	3.77^a^ ± 0.19	4.22^b^ ± 0.1	4.42^b^ ± 0.13	5.35^c^ ± 0.18	5.02^d^ ± 0.23

Results are shown as mean ± SD of at least five individual plants. Different letters denote significant differences at *p* ≤ 0.05 analyzed by Tukey's test.
