# Supplementary material for: A basic helix-loop-helix transcription factor, PhFBH4, regulates flower senescence by modulating ethylene biosynthesis pathway in petunia
Source: Hortic Res. 2015 Dec 16;2:15059–. doi: 10.1038/hortres.2015.59 (PMC4680862; doi:10.1038/hortres.2015.59)
Supplement: Supplementary Information [file hortres201559-s1.docx]

**A Basic Helix-Loop-Helix Transcriptional Factor, *PhFBH4*, Regulates Flower Senescence by Mediating Ethylene Biosynthesis in Petunia**

Jing Yin, Xiaoxiao Chang, Takao Kasuga, Mai Bui, Michael S. Reid, Cai-Zhong Jiang


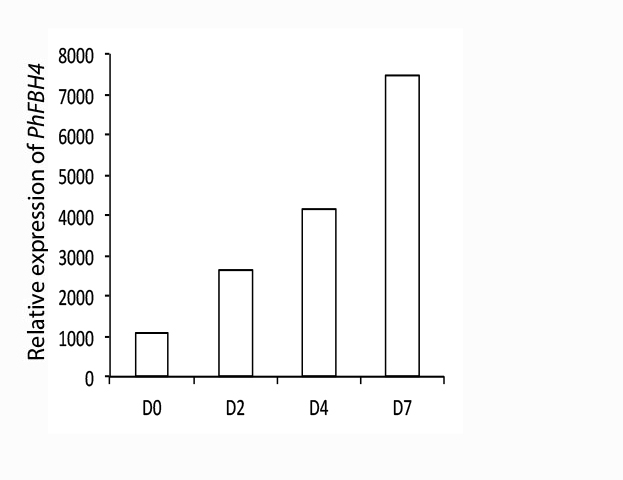


Figure S1. *PhFBH4* transcript abundance during petunia flower senescence using a custom-designed microarray (NimbleGen). D0: the day of anthesis, D2, D4, D6, D7: 2, 4, 6 and 7 days after anthesis, respectively.


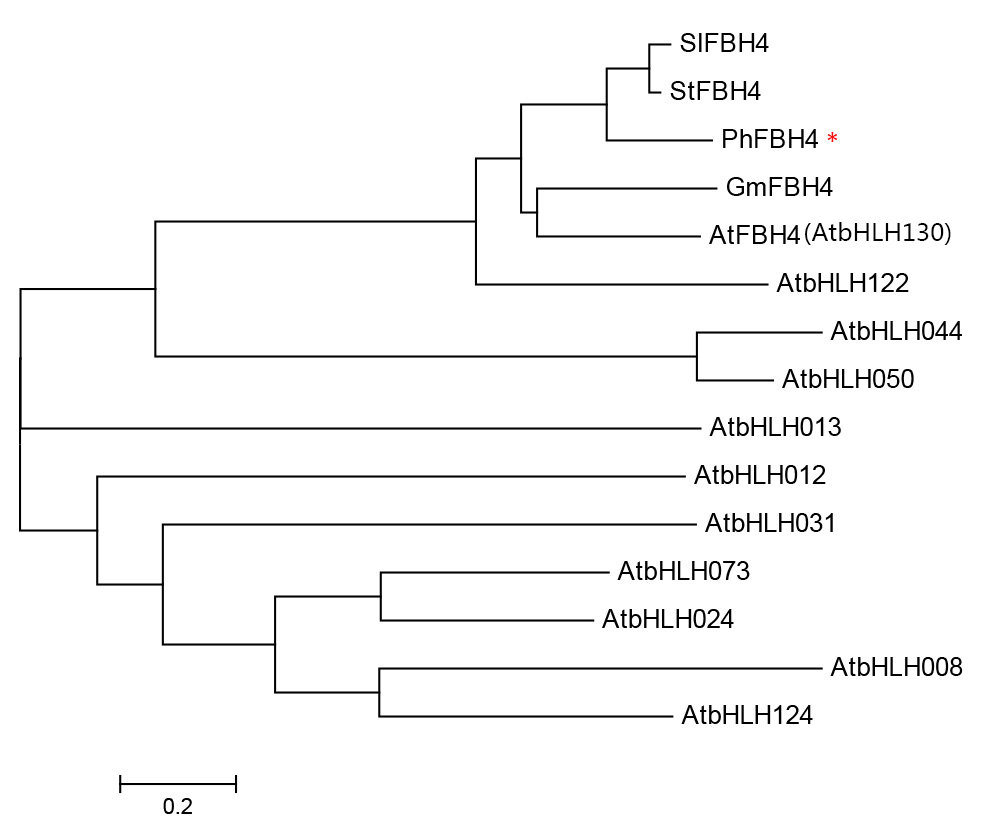


Figure S2. Phylogenetic analysis of deduced PhbHLH amino acid sequence with bHLH proteins from other plant species. The phylogenetic tree file was produced by MEGA 5.2.


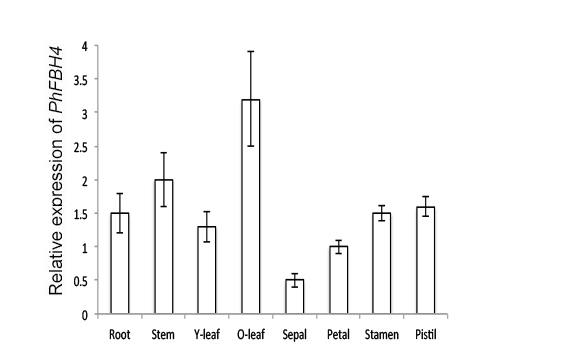


Figure S3. *PhFBH4* transcripts in different tissues. The young and old leaves were collected at the 3rd and 9th leaves from the top of the branches separately. The stems were collected between the 2nd and the 3rd leaves from the top of the branches. Flower tissue was collected from flower on D0 before anther dehiscence.

Table S1 Oligonucleotide primer sequences.

| Primers | Sequence |
| --- | --- |
| PhFBH4-Full length-UP | ATTGGATCCATGGAGTCAGAATTTG |
| PhFBH4-Full length-LOW | ATTACTAGTTCAGACTCTTTGGTTG |
| PhFBH4-antisense-UP | ACGGGATCCTTGGTAAACTTGAGTGGTGTG |
| PhFBH4-antisense-LOW | ATTACTAGTTCCCATCAGGGGCACCTAC |
| PhFBH4-VIGS-UP | AGCGAATTCAATCAGTGAAAGAATGAGG |
| PhFBH4-VIGS-LOW | AGCTCTAGAGTTGTTTGAGACCTTCTTGG |
| PhFBH4-qRTPCR-UP | ATTCCATGGTCCCATCAGGGGCACCTAC |
| PhFBH4-qRTPCR-UP | CGGGGCGCGCCTTGGTAAACTTGAGTGGTG |
| CHS-UP | AACCATTGGGCATTTCTG |
| CHS-LOW | AGCCTTTCTCATTTCATTC |
| 26S-UP | AGCTCGTTTGATTCTGATTTCCAG |
| 26S-LOW | GATAGGAAGAGCCGACATCGAAGG |
| SAG12-UP | GTACAAGGGAGAAGACGGTGTC |
| SAG12-LOW | GTACAAGGGAGAAGACGGTGTC |
| SAG29-UP | TACGCCACCAGGGAGAAAAGGAT |
| SAG29-LOW | GGCAGCGAAAACAGAAACAGAAATG |
| ACO1-UP | GGATCCAATTGCCACTGTCTA |
| ACO1-LOW | CTGATGGAGAAATGAAGGAACA |
| ACO4-UP | AGAGTGTACCGCACAGAGTGATT |
| ACO4-LOW | GATCAATCTTCACATCAGCTTCC |
| ACS1-UP | CAAGAGGGGGTAGGGTTAGC |
| ACS1-LOW | GAGGTGGATGTTTCGTTCGT |
| ACS2-UP | CACGTGTAATTCGCATGGAC |
| ACS2-LOW | GGTGAGGACGAGGTTGTTTG |
| ACS3-UP | GCCTATTTTGATGGCTGGAA |
| ACS3-LOW | CTCCAGTTGCTCCTCCACTC |
| GA20OX1-UP | GCAGCCCAACAAGCATCTAC |
| GA20OX1-LOW | AGTGTCTCTTTCCAAGGTAGCT |
| GA20OX2-UP | TTGATGAAGCATGCAGGAAC |
| GA20OX2-LOW | CCTTCGGCAGAATATGGAAA |
| GA2OX1-UP | TGAGATCCAACAACACTTCTGG |
| GA2OX1-LOW | CCATCGTTCCTAACAAGATTCC |
| GA2OX3-UP | GAACACACAGACCCTCAAATCA |
| GA2OX-LOW | GCAACTGTCTCAAATAGGACCA |
